# A combined index of waist circumference and muscle quality is associated with cardiovascular disease risk factor accumulation in Japanese obese patients: a cross-sectional study

**DOI:** 10.1007/s12020-022-03052-5

**Published:** 2022-04-19

**Authors:** Kentaro Ikeue, Toru Kusakabe, Kazuya Muranaka, Hajime Yamakage, Takayuki Inoue, Kojiro Ishii, Noriko Satoh-Asahara

**Affiliations:** 1 Department of Endocrinology, Metabolism, and Hypertension Research, Clinical Research Institute, National Hospital Organization Kyoto Medical Center, 1-1 Fukakusa Mukaihata-cho, Fushimi-ku, Kyoto 612-8555 Japan; 2 Graduate School of Health and Sports Science, Doshisha University, 1-3 Tatara Miyakodani, Kyotanabe, Kyoto 610-0394 Japan; 3 Faculty of Health and Sports Science, Doshisha University, 1-3 Tatara Miyakodani, Kyotanabe, Kyoto 610-0394 Japan

**Keywords:** Obesity, Sarcopenia, Sarcopenic obesity, Waist circumference, Muscle quality, Cardiovascular disease

## Abstract

**Purpose:**

To identify obese patients at high risk of cardiovascular disease (CVD) using a combined index of obesity and sarcopenia.

**Methods:**

In this cross-sectional study, we firstly conducted analysis of covariance to select each index most associated with the CVD risk score, the number of concomitant CVD risk factors, among obesity- (body mass index, percentage body fat, or waist circumference [WC]) and sarcopenia-evaluated indices (skeletal muscle mass index, handgrip strength, or muscle quality [MQ]), respectively in 188 Japanese obese patients (BMI ≥ 25 kg/m^2^, 73 men and 115 women). Next, we conducted multivariate logistic regression analysis to compare the four groups (Group A–D) classified by medians of the selected indices.

**Results:**

WC and MQ were selected as the indices most associated with the CVD risk scores, respectively. The CVD risk score was significantly higher in Group B (low WC and low MQ) and Group D (high WC and low MQ) with higher prevalence of diabetes as compared with Group A (low WC and high MQ). Adjusted for sex and age, odds ratios for CVD risk scores = 2 were significantly higher in Group B, Group C (high WC and high MQ), and Group D compared with Group A. Furthermore, odds ratios for CVD risk scores = 3 were significantly higher only in Group D compared with Group A (4.29 [95% confidence interval: 1.49–12.33], *p* = 0.007).

**Conclusion:**

Combined index of WC and MQ was useful in Japanese obese patients at high risk of CVD, regardless sex and age.

## Introduction

Aging induces changes in body composition,such as an increase in body fat and a decline in skeletal muscle [1, 2]. Body fat increases until the seventh decade of life and decreases thereafter [3]. It has been reported that most of the body fat increase with aging is due to the increase in visceral fat (VF) [4]. On the other hand, skeletal muscle mass and strength reach their maximum amount at young adulthood (up to ~40 years of age) and then decline by several percent each year [5].

Sarcopenia is the loss of muscle mass and strength or physical function that occurs naturally with aging [3, 5, 6]. Probable sarcopenia is identified by low muscle strength, and the diagnosis of sarcopenia is confirmed by the additional documentation of low muscle quantity or quality [5]. According to a recent systematic review and meta-analysis, the worldwide prevalence of sarcopenia is 10% (95% confidence interval [CI] 8–12%) in men and 10% (95% CI 8–13%) in women, respectively [7]. It has been reported that sarcopenia is associated with a number of different outcomes such as falls and fractures [8–10], disability [8, 11], metabolic syndrome [12], CVD [13, 14], and mortality [8, 15].

Sarcopenic obesity (SO) was first described by Heber et al. as the co-presence of sarcopenia and obesity [16]. Sarcopenia and obesity have some common pathophysiological mechanisms, including increased inflammatory cytokines, oxidative stress, insulin resistance, hormonal changes, and decreased physical activity [1]. Furthermore, a vicious cycle may exist between sarcopenia and obesity; that is, sarcopenia reduces physical activity, leading to an increase in the risk of obesity, and excess accumulation of VF induces inflammation, leading to the development of sarcopenia. Therefore, it is feared that sarcopenic obesity will increase with aging.

Elderly individuals with SO have higher risks of low physical function [17, 18], metabolic diseases [19, 20], CVD [21–23], and mortality [21, 24]. These clinical problems in SO are much more severe than in sarcopenia or obesity alone. In the diagnosis of SO, sarcopenia and obesity have been diagnosed separately as two distinct categories. However, worldwide diagnostic criteria for SO and its cutoff values have not yet been established [25, 26]. One probable reason for the difficulty in establishing diagnostic criteria for SO is that there are multiple ways to measure body composition. Magnetic resonance imaging (MRI) and computed tomography (CT) are considered to be gold standards for non-invasive assessment of muscle mass [27]. However, these tools are not commonly in primary care because of high equipment costs, lack of portability [27]. Dual-energy X-ray absorptiometry (DXA) is a more widely available instrument to muscle mass, however not yet portable for use [5]. Recently, bioelectrical impedance analysis device (BIA) is affordable, widely available and portable [5]. Body composition measured using a multifrequency BIA was highly correlated with measurements obtained from of DXA [28]. Another probable reason is that there are multiple combinations for evaluating sarcopenia and obesity. For example, Kim et al. diagnosed SO by skeletal muscle mass index (SMI) and high percentage body fat (PBF) and investigated its association with metabolic syndrome [29]. In addition, Schrager et al. reported that sarcopenic obesity diagnosed using body mass index (BMI), waist circumference (WC) and handgrip strength (HGS) was associated with elevated proinflammatory, especially central obesity and low HGS [30]. The diagnostic criteria for SO needs to be considered for each of the different subjects and clinical outcomes.

In this study, to identify obese patients at high risk of CVD, we examined a combined index most associated with CVD risk factor accumulation among obesity-evaluated indices, BMI, PBF, or WC, and sarcopenia-evaluated indices, SMI, HGS, or muscle quality (MQ) in Japanese obese patients. We then classified obese patients into four groups using medians of the two selected indices and compared the CVD risk score.

## Methods

### Study participants

This cross-sectional study included obese outpatients who regularly visited the Diabetes Center at the National Hospital Organization Kyoto Medical Center between January 2019 and July 2019. The diagnosis of obesity was based on the standards of Japan Society for the Study of Obesity, BMI ≥ 25 kg/m^2^ [31]. We uniformly provided exercise and dietary guidance for all obese outpatients in accordance with the guidelines of the Japan Society for the study of obesity. We excluded participants with incomplete data, implantation of a cardiac pacemaker, and cancer from the study. None of the patients had sarcopenia secondary to CVDs, respiratory diseases, endocrinological diseases or conditions of secondary obesity such as Cushing’s syndrome. This study was approved by the Ethics Committee for Human Research at National Hospital Organization Kyoto Medical Center (approval No. 19-083) and was conducted in accordance with the principles of the Declaration of Helsinki and the ethical guidelines for medical and health research involving human subjects.

### Clinical examination

We measured height and body weight in increments of 0.1 cm and 0.1 kg, respectively. BMI was calculated as the body weight (kg) divided by the squared height (m^2^). WC was measured at the umbilical level in a standing position. HGS was measured twice for each hand using the Smedley grip force system (Grip-D, Takei Equipment Company, Tokyo, Japan) in a standing position, and the maximum value was included in the analyses. The appendicular skeletal muscle mass (ASM) and PBF were measured using a multifrequency BIA (MC-780A-N, TANITA, Tokyo, Japan). SMI was calculated as the ASM (kg) divided by the squared height (m^2^). In this study, MQ was calculated as the HGS (kg) divided by muscle mass of the upper limbs (kg) according to previous reports [32–34]. Systolic and diastolic blood pressure was measured with an automatic electrical sphygmomanometer (BP-203RVII, Fukuda Colin, Kyoto, Japan). Blood was taken from the antecubital vein in the morning after an overnight fast, and we determined fasting plasma glucose, hemoglobin A1c (HbA1c), triglycerides (TG), high-density lipoprotein cholesterol (HDL-C), and low-density lipoprotein cholesterol (LDL-C).

### Diagnosis of sarcopenia

Sarcopenia was diagnosed by low SMI and weak HGS [6]. The cutoff values for low SMI were <7.0 kg/m^2^ for men and <5.7 kg/m^2^ for women and those for weak HGS were <28 kg for men and <18 kg for women according to the guideline of Asian Working Group for Sarcopenia, respectively [6].

### Diagnosis of hypertension, diabetes, and dyslipidemia

The diagnosis of hypertension, diabetes, and dyslipidemia was based on the criteria of each academic society; hypertension was defined as systolic blood pressure ≥140 mmHg and/or diastolic blood pressure ≥90 mmHg or taking medications for hypertension [35]; diabetes was defined by fasting plasma glucose ≥126 mg/dL, and/or HbA1c (National Glycohemoglobin Standardization Program) ≥6.5%, or taking medications for diabetes [36]; dyslipidemia was defined by LDL-C ≥ 140 mg/dL, and/or HDL-C < 40 mg/dL, and/or TG ≥ 150 mg/dL, or taking medications for dyslipidemia [37].

### Definition of CVD risk score

In this study, the CVD risk score was defined as the number of concomitant CVD risk factors (hypertension, diabetes, and dyslipidemia; 0–3 points), referring to a previous report [38].

### Classification of obese patients using each obesity- and sarcopenia-evaluated index

**Fig. 1:**
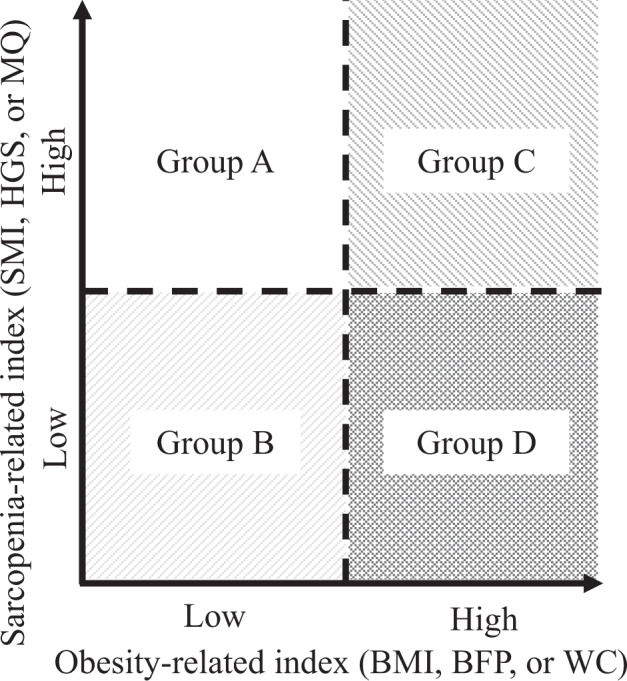
Classification using each obesity- and sarcopenia-evaluated index. Obese patients were classified into four groups (Group A, Group B, Group C, and Group D) using medians of each obesity- and sarcopenia-evaluated index

As shown in Fig. 1, obese patients were classified into four groups using each obesity- and sarcopenia-evaluated index with the median value; Group A, low obesity-evaluated index and high sarcopenia-evaluated index; Group B, low obesity-evaluated index and low sarcopenia-evaluated index; Group C, high obesity-evaluated index and high sarcopenia-evaluated index; Group D, high obesity-evaluated index and low sarcopenia-evaluated index.

### Statistical analysis

We performed statistical analyses using SPSS (version 25; IBM Corp, Armonk, NY, USA). Data are presented as mean ± standard deviation, median (interquartile range [IQR]), or frequency percentage. In all cases, a probability (*p*) value of < 0.05 was considered statistically significant.

Obese patients were dichotomized by the median value in each obesity- and sarcopenia-evaluated index. Those above the median value were classified as “high” and those below as “low”. We conducted analysis of covariance (ANCOVA) to compare the CVD risk scores between the low and high groups in each obesity-evaluated index (BMI, PBF, or WC) and sarcopenia-evaluated index (SMI, HGS, or MQ). ANCOVA was constructed as follows: model 1 was unadjusted, model 2 was adjusted for sex, and model 3 was further adjusted for age. Next, we conducted analysis of variance (ANOVA) followed by Tukey’s test or Kruskal–Wallis test followed by Bonferroni correction for continuous variables and chi-square test for categorical variables to compare the characteristics of the four groups (Group A–D). We then conducted ANCOVA followed by Bonferroni correction to compare the CVD risk scores among the four groups. Lastly, to examine the association between severity of CVD risk factors and the combined index, we used multiple logistic regression analysis adjusted for sex and age to determine odds ratios (ORs) and 95% clinical intervals (CIs) for each CVD risk score (=1, =2, and =3) as compared with Group A.

## Results

### Clinical characteristics of the study participants

**Fig. 2:**
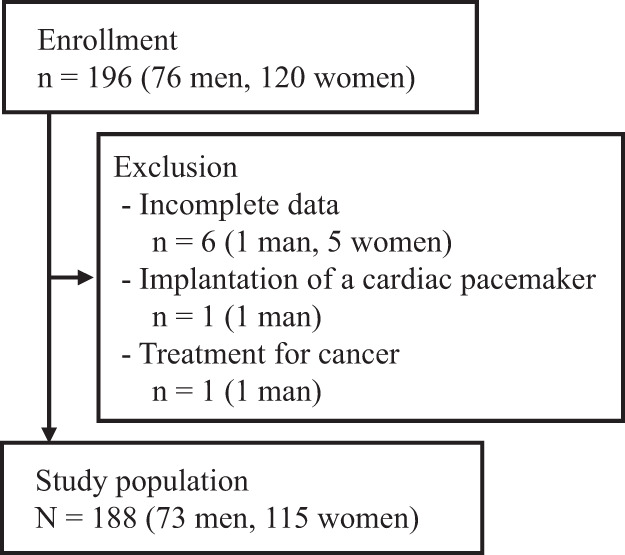
Study flow chart of participants

As shown in Fig. 2, 196 patients (76 men and 120 women) were enrolled in this study. Six patients with incomplete data, one patient with a cardiac pacemaker, and one patient undergoing treatment for cancer were excluded. Finally, 188 Japanese obese patients (73 men and 115 women) were included in the study population.

Table 1 summarizes the clinical characteristics of the obese patients. Elderly patients aged 65 years and older included 22 men (30.1%) and 40 women (34.8%), respectively. As for the obesity-evaluated indices, BMIs were 30.6 (IQR: 27.3–34.2) kg/m^2^ in men and 31.8 (IQR: 28.8–36.3) kg/m^2^ in women, PBF values were 34.3% ± 7.2% in men and 48.5% ± 8.0% in women, and WCs were 103.0 (IQR: 98.5–111.0) cm in men and 102.0 (IQR: 95.0–111.0) cm in women, respectively. There were 71 men (97.3%) with an abdominal circumference of ≥85 cm and 102 women (88.7%) with an abdominal circumference of ≥90 cm, suggesting that the participants had substantial VF accumulation. On the other hand, as for the sarcopenia-evaluated indices, SMIs were 8.94 ± 1.04 kg/m^2^ in men and 7.47 ± 0.83 kg/m^2^ in women, HGS values were 39.6 ± 7.4 kg in men and 23.7 ± 4.9 kg in women, and MQs were 6.59 ± 1.12 kg/kg in men and 6.52 ± 1.18 kg/kg in women, respectively. Low SMI was observed in 3 men (4.1%) and weak HGS was noted in 5 men (6.8%) and 14 women (12.2%), respectively. However, sarcopenia was diagnosed in only one male (1.4%). The prevalence of hypertension, diabetes, and dyslipidemia was high; therefore, patients had the high CVD risk scores (1.92 ± 0.86 in men and 1.65 ± 0.90 in women).

**Table 1:** Clinical characteristics of the obese patients

	All (*n* = 188)	Male (*n* = 73)	Female (*n* = 115)
Age (year)	55.7 ± 15.7	54.7 ± 16.7	56.4 ± 15.1
BMI (kg/m^2^)	31.2 (28.1–35.0)	30.6 (27.3–34.2)	31.8 (28.8–36.3)
PBF (%)	43.0 ± 10.3	34.3 ± 7.2	48.5 ± 8.0
WC (cm)	102.0 (97.0–111.0)	103.0 (98.5–111.0)	102.0 (95.0–111.0)
SMI (kg/m^2^)	8.04 ± 1.16	8.94 ± 1.04	7.47 ± 0.83
HGS (kg)	29.9 ± 9.8	39.6 ± 7.4	23.7 ± 4.9
MQ (kg/kg)	6.55 ± 1.16	6.59 ± 1.12	6.52 ± 1.18
SBP (mmHg)	136.0 ± 13.6	138.0 ± 13.8	134.8 ± 13.4
DBP (mmHg)	82.1 ± 9.5	83.3 ± 9.0	81.3 ± 9.8
FPG (mg/dl)	116.2 ± 32.3	117.9 ± 28.0	115.2 ± 34.8
HbA1c (%)	6.4 ± 1.2	6.4 ± 1.2	6.4 ± 1.3
TG (mg/dL)	133.4 ± 76.2	138.8 ± 83.3	129.9 ± 71.4
HDL-C (mg/dL)	57.5 ± 14.9	52.4 ± 11.5	60.7 ± 15.9
LDL-C (mg/dL)	118.8 ± 28.1	114.8 ± 23.8	121.4 ± 30.4
Current smoker (%)	8.5	12.3	6.1
Hypertension (under treatment) (%)	68.1 (43.1)	74.0 (45.2)	64.3 (41.7)
Medications for hypertension (*n*) (CA/ACEI/ARB/diuretics/β/αβ/DRI)	68/8/57/19/6/2/1	32/4/24/6/4/2/0	36/4/33/13/2//0/1
Diabetes (under treatment) (%)	36.7 (28.2)	43.8 (31.5)	32.2 (26.1)
Medications for diabetes (*n*) (SU/DPP4I/BG/SGLT2I/GLI/αGI/insulin)	22/35/27/21/1/2/8	10/14/12/10/1/2/4	12/21/15/11/0/0/4
Dyslipidemia (under treatment) (%)	70.7 (45.7)	74.0 (56.2)	68.7 (39.1)
Medications for dyslipidemia (*n*) (statin/fibrate/ω3)	67/3/25	30/1/14	37/2/11
CVD risk score	1.76 ± 0.89	1.92 ± 0.86	1.65 ± 0.90

Data are mean ± SD, or median (interquartile range), or frequency percentage

*BMI* body mass index, *PBF* percentage body fat, *WC* waist circumference, *SMI* skeletal muscle mass index, *HGS* handgrip strength, *MQ* muscle quality, *SBP* systolic blood pressure, *DBP* diastolic blood pressure, *FPG* fasting plasma glucose, *HbA1c* hemoglobin A1c, *TG* triglyceride, *HDL-C* high-density lipoprotein cholesterol, *LDL-C* low-density lipoprotein cholesterol, *CA* calcium channel antagonist, *ACEI* ACE inhibitor, *ARB* angiotensin receptor blocker, *β* β-blockade, *αβ* αβ-blockade, *DRI* direct renin inhibitor, *SU* sulfonyl urea, *DPP4I* dipeptidyl peptidase–4 inhibitor, *BG* biguanide, *SGLT2I* sodium glucose cotransporter 2 inhibitor, *GLI* glinide, *αGI* alpha glucosidase inhibitor, *CVD* cardiovascular disease

### Obesity-evaluated index most associated with CVD risk factor accumulation

For the obesity-evaluated indices, the median BMI was 30.6 kg/m^2^ in men and 31.8 kg/m^2^ in women, the median PBF was 34.1% in men and 47.7% in women, and the median WC was 103.0 cm in men and 102.0 cm in women, respectively. Table 2A shows the comparisons of the CVD risk scores between the low and high groups in each obesity-evaluated index. The CVD risk score was significantly higher in the high group as compared with the low group only for WC (1.62 [95% CI: 1.43–1.80] vs. 1.88 [95% CI: 1.70–2.05], *p* < 0.05; model 1). Furthermore, the association persisted even after adjusting for sex (1.61 [95% CI: 1.43–1.80] vs. 1.88 [95% CI: 1.71–2.06], *p* < 0.05; model 2) and for sex and age (1.58 [95% CI: 1.40–1.76] vs. 1.91 [95% CI: 1.74–2.08], *p* < 0.01; model 3).

**Table 2:** Comparisons of the CVD risk scores between the low and high groups in each index

A. Obesity-evaluated indices												
	BMI				PBF				WC			
	Low (*n* = 93)		High (*n* = 95)		Low (*n* = 93)		High (*n* = 95)		Low (*n* = 89)		High (*n* = 99)	
Model 1	1.73	(1.55, 1.91)	1.78	(1.60, 1.96)	1.66	(1.48, 1.84)	1.85	(1.67, 2.03)	1.62	(1.43, 1.80)	1.88*	(1.70, 2.05)
Model 2	1.73	(1.55, 1.91)	1.78	(1.60, 1.96)	1.66	(1.48, 1.84)	1.85	(1.67, 2.03)	1.61	(1.43, 1.80)	1.88*	(1.71, 2.06)
Model 3	1.64	(1.46, 1.83)	1.87	(1.68, 2.05)	1.62	(1.44, 1.79)	1.89*	(1.72, 2.07)	1.58	(1.40, 1.76)	1.91**	(1.74, 2.08)

B. Sarcopenia-evaluated indices												
	SMI				HGS				MQ			
	Low (*n* = 93)		High (*n* = 95)		Low (*n* = 92)		High (*n* = 96)		Low (*n* = 93)		High (*n* = 95)	
Model 1	1.77	(1.59, 1.96)	1.74	(1.56, 1.92)	1.80	(1.62, 1.99)	1.71	(1.53, 1.89)	1.95**	(1.77, 2.13)	1.57	(1.40, 1.75)
Model 2	1.78	(1.59, 1.96)	1.74	(1.56, 1.92)	1.80	(1.62, 1.99)	1.71	(1.53, 1.89)	1.95**	(1.77, 2.12)	1.57	(1.39, 1.74)
Model 3	1.65	(1.46, 1.85)	1.86	(1.67, 2.04)	1.71	(1.53, 1.90)	1.80	(1.61, 1.98)	1.93**	(1.76, 2.10)	1.58	(1.41, 1.75)

Data are estimated mean (95% CIs)

*BMI* body mass index, *PBF* percentage body fat, *WC* waist circumference, *SMI* skeletal muscle mass index, *HGS* handgrip strength, *MQ* muscle quality

Model 1 unadjusted, Model 2 adjusted for sex, Model 3 adjusted for sex and age

*p < 0.05, **p < 0.01 by ANCOVA between the low group vs. the high group in each obesity- and sarcopenia-evaluated index

### Sarcopenia-evaluated index most associated with CVD risk factor accumulation

For the sarcopenia-evaluated indices, the median SMI was 8.98 kg/m^2^ in men and 7.37 kg/m^2^ in women, the median HGS was 40.6 kg in men and 23.5 kg in women, and the median MQ was 6.63 kg/kg in men and 6.58 kg/kg in women, respectively. Table 2B shows the comparisons of the CVD risk scores between the low and high groups in each sarcopenia-evaluated index. The CVD risk score was significantly higher in the low group compared with the high group only for MQ (1.95 [95% CI: 1.77–2.13] vs. 1.57 [95% CI: 1.40–1.75], *p* < 0.01; model 1). Furthermore, the association persisted even after adjusting for sex (1.95 [95% CI: 1.77–2.12] vs. 1.57 [95% CI: 1.39–1.74], *p* < 0.01; model 2) and for sex and age (1.93 [95% CI: 1.76–2.10] vs. 1.58 [95% CI: 1.41–1.75], *p* < 0.01; model 3).

### Clinical characteristics of the obese patients classified by a combined index of WC and MQ

Based on the above results, we selected WC and MQ as the indices most associated with CVD risk factor accumulation, respectively. Then, we classified obese patients into four groups (Group A, low WC and high MQ; Group B, low WC and low MQ; Group C, high WC and high MQ; and Group D, high WC and low MQ) (Fig. 1). By this classification, Group B had significantly lower MQ compared with Group A (5.49 ± 0.85 vs. 7.50 ± 0.71 kg/kg, *p* < 0.05), Group C had significantly higher WC compared with Group A (111.0 [IQR: 106.3–118.3] vs. 95.0 [IQR: 89.8–99.0] cm, *p* < 0.05), and Group D had significantly lower MQ and higher WC compared with Group A (5.71 ± 0.72 vs. 7.50 ± 0.71 kg/kg, *p* < 0.05; 110.5 [IQR: 106.8–121.5] vs. 95.0 [IQR: 89.8–99.0] cm, *p* < 0.05, respectively; Table 3). As for other obesity- and sarcopenia-evaluated indices, both BMI and PBF were also significantly higher in Group C and Group D compared with Group A (all *p* < 0.05), and HGS was also lower in Group B and Group D compared with Group A (all *p* < 0.05; Table 3). Interestingly, in contrast to MQ, the SMI in Group D was significantly higher than that in Group A (8.32 ± 1.27 vs. 7.82 ± 1.03 kg/m^2^, *p* < 0.05).

**Table 3:** Clinical characteristics of the obese patients classified by a combined index of WC and MQ

	Group A (*n* = 54)	Group B (*n* = 35)	Group C (*n* = 41)	Group D (*n* = 58)	*p* value^§^
Age (year)	55.5 ± 14.5	62.1 ± 12.2	53.1 ± 16.5	53.8 ± 17.4	0.049
Women (%)	57.4	62.9	65.9	60.3	0.859
BMI (kg/m^2^)	27.6 (26.1–30.3)	28.7 (26.9–30.3)	34.5 (31.9–39.9)*†	34.2 (32.1–39.9)*†	<0.001
PBF (%)	36.9 ± 8.8	38.4 ± 7.0	48.4 ± 9.9*†	47.6 ± 9.5*†	<0.001
WC (cm)	95.0 (89.8–99.0)	97.0 (92.0–100.0)	111.0 (106.3–118.3)*†	110.5 (106.8–121.5)*†	<0.001
SMI (kg/m^2^)	7.82 ± 1.03	7.72 ± 1.13	8.22 ± 1.10†	8.32 ± 1.27*†	0.029
HGS (kg)	33.0 ± 9.7	25.1 ± 9.6*	32.6 ± 9.1†	27.9 ± 9.0*#	<0.001
MQ (kg/kg)	7.50 ± 0.71	5.49 ± 0.85*	7.38 ± 0.55†	5.71 ± 0.72*#	<0.001
SBP (mmHg)	132.5 ± 12.5	133.7 ± 14.9	139.4 ± 14.2*†	138.3 ± 12.7*	0.036
DBP (mmHg)	83.1 ± 9.8	80.2 ± 9.8	84.9 ± 7.1	80.3 ± 10.2	0.063
FPG (mg/dL)	108.7 ± 31.3	117.9 ± 31.4	124.9 ± 31.5	126.1 ± 46.4	0.061
HbA1c (%)	6.0 ± 0.8	6.3 ± 0.8	6.6 ± 1.4	6.6 ± 1.5	0.028
TG (mg/dL)	134.0 ± 96.2	126.9 ± 56.0	136.3 ± 73.4	134.5 ± 68.9	0.956
HDL-C (mg/dL)	59.2 ± 13.9	56.8 ± 16.0	57.3 ± 13.2	56.5 ± 16.4	0.794
LDL-C (mg/dL)	116.7 ± 26.7	119.9 ± 28.6	123.9 ± 26.7	116.4 ± 30.1	0.526
Current smoker (%)	3.7	14.3	12.2	6.9	0.260
Hypertension (%)	57.4	68.6	70.7	75.9	0.205
Diabetes (%)	16.7	48.6*	41.5*	44.8*	0.004
Dyslipidemia (%)	66.7	77.1	65.9	74.1	0.589
CVD risk score	1.41 ± 0.84	1.94 ± 0.80*	1.78 ± 0.91	1.95 ± 0.91**	0.005

Data are mean ± SD or median (interquartile range), or frequency percentage

*BMI* body mass index, *PBF* percentage body fat, *WC* waist circumference, *SMI* skeletal muscle mass index, *HGS* handgrip strength, *MQ* muscle quality, *SBP* systolic blood pressure, *DBP* diastolic blood pressure, *FPG* fasting plasma glucose, *HbA1c* hemoglobin A1c, *TG* triglyceride, *HDL-C* high-density lipoprotein cholesterol, *LDL-C* low-density lipoprotein cholesterol, *CVD* cardiovascular disease

^§^*p* value for difference among the four groups in means (ANOVA followed by Tukey’s test), or medians (Kruskal–Wallis test followed by Bonferroni correction), or percentages (Chi-square test)

*p < 0.05 vs. Group A, ^†^*p* < 0.05 vs. Group B, ^#^*p* < 0.05 vs. Group C

Although there was no significant difference in the prevalence of hypertension and dyslipidemia among the four groups, that of diabetes was significantly higher in Group B, Group C, and Group D compared with Group A (48.6, 41.5, 44.8 vs. 16.7%, all *p* < 0.05; Table 3). The CVD risk score was also significantly higher in Group B and Group D compared with Group A (1.94 ± 0.80, 1.95 ± 0.91 vs. 1.41 ± 0.84, all *p* < 0.05; Table 3). In addition, Table 4 shows the multiple comparisons of CVD risk scores among the four groups. Even in the model 2, adjusted for sex, the significant differences between Group B, D and Group A (1.95 [95%CI 1.66, 2.23], 1.95 [95%CI 1.72, 2.17] vs. 1.40 [95%CI 1.17, 1.63], all *p* < 0.05) were retained. However, in the model 3, adjusted for sex and age, the significant difference between Group D and Group A was retained (1.97 [95%CI 1.77, 2.19] vs. 1.40 [95%CI 1.17, 1.62], *p* < 0.05), although between Group B and Group A was not (1.87 [95%CI 1.59, 2.16], *p* = 0.067).

**Table 4:** Comparison of CVD risk scores in obese patients classified by a combined index of WC and MQ

	Group A		Group B		Group C		Group D	
Model 1	1.41	(1.17, 1.64)	1.94*	(1.65, 2.23)	1.78	(1.51, 2.05)	1.95**	(1.72, 2.17)
Model 2	1.40	(1.17, 1.63)	1.95*	(1.66, 2.23)	1.79	(1.53, 2.06)	1.95**	(1.72, 2.17)
Model 3	1.40	(1.17, 1.62)	1.87	(1.59, 2.16)	1.83	(1.57, 2.09)	1.97**	(1.75, 2.19)

Data are estimated mean (95% CIs)

*CVD* cardiovascular disease, *WC* waist circumference, *MQ* muscle quality

Model 1 unadjusted, model 2 adjusted for sex, model 3 adjusted for sex and age

*p < 0.05, **p < 0.01 by ANCOVA followed by Bonferroni correction vs. group A

### ORs for CVD risk factor accumulation in obese patients classified by a combined index of WC and MQ

**Fig. 3:**
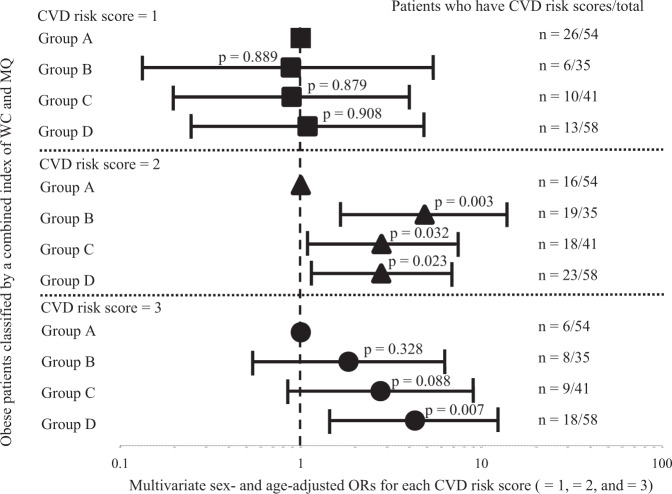
ORs for the CVD risk scores in obese patients of each group. Multivariate sex- and age-adjusted ORs in obese patients classified by a combined index of WC and MQ (Group A, Group B, Group C, and Group D). Squares, ORs for CVD risk score = 1; triangles, ORs for CVD risk score = 2; circles, ORs for CVD risk score = 3. Solid horizontal lines, 95% CIs

Finally, we investigated the effect of a combined index of WC and MQ on the CVD risk score (Fig. 3). We found no difference in ORs for CVD risk scores = 1 among the four groups. However, ORs for CVD risk scores = 2 were significantly higher in Group B, Group C, and Group D compared with Group A (4.85 [95% CI: 1.72–13.72], *p* = 0.003; 2.80 [95% CI: 1.09–7.18], *p* = 0.032; 2.79 [95% CI: 1.15–6.74], *p* = 0.023; respectively). Furthermore, ORs for CVD risk scores = 3 were significantly higher only in Group D compared with Group A (4.29 [95% CI: 1.49–12.33], *p* = 0.007).

## Discussion

To the best of our knowledge, this is the first study to determine a combined index associated with CVD risk factor accumulation in Japanese obese patients among each of the obesity- (BMI, PBF, or WC) and sarcopenia-evaluated indices (SMI, HGS, or MQ), respectively. Among obese patients, sarcopenia was diagnosed in only one man (1.4%). This result is consistent with previous studies reporting that few obese individuals diagnosed with high BMI meet the conventional diagnostic criteria for sarcopenia [5, 6, 39]. However, the prevalence of hypertension, diabetes, and dyslipidemia in these patients was high (Table 1), indicating that they are at high risk for CVD. Therefore, we attempted to identify obese patients at high risk of cardiovascular disease (CVD) using a combined index of obesity and sarcopenia, such as SO. In this study, we provided evidence that classification using the combined index of WC and MQ reflects CVD risk factor accumulation in Japanese obese patients, regardless sex and age.

Many indices have been proposed for evaluating obesity. However, controversy remains on the obesity-evaluated index that best reflects CVD risk factor accumulation [40]. In this study, WC was most highly associated with CVD risk factor accumulation as compared with BMI and PBF, regardless of sex and age (Table 2A). BMI has been widely used to determine the prevalence of obesity and various risks in populations. In addition, PBF is often used as a criterion for evaluating the magnitude of accumulation of adipose tissue. However, in recent years, the indices of abdominal obesity, mainly WC, have been shown to be more closely related to CVD and mortality than BMI and PBF are [41, 42] which is consistent with the findings of our study.

Many indices have been proposed to evaluate sarcopenia, including muscle mass, muscle strength, physical performance, and MQ, but it is also unclear which sarcopenia-evaluated index best reflects CVD risk accumulation. Cao et al. reported that in patients with metabolic syndrome, low SMI may be an independent risk factor for atherosclerosis [43]. In addition, low HGS has been reported to be associated with coronary artery calcification, CVD, and all-cause mortality [44–46]. Our study demonstrated that MQ was most associated with CVD risk factor accumulation compared with SMI and HGS, regardless of sex and age (Table 2B). MQ was reported to be negatively associated with insulin resistance after adjusting for age, body fat, highly sensitive C-reactive protein levels, and physical activity level in adult obese women [47] and further that insulin resistance contributes to the development of atherosclerosis [48, 49], findings that are consistent with our results.

In this study, HGS was lower in Group D compared with Group A, whereas SMI was significantly higher in Group D compared with Group A. Therefore, MQ calculated as the HGS divided by the muscle mass of the upper limbs was significantly lower in Group D compared with Group A. Mesinovic et al. reported that overweight and obese older adults with metabolic syndrome have larger muscle size but poor MQ [50]. The lower MQ in Group D might be attributed to increased fat accumulation in the muscles [51, 52]; however, in this study, we did not measure MQ with imaging analysis, such as ultrasonography or computed tomography. Recently, the usefulness of phase angle as an indicator for MQ has been suggested [5, 53], and further studies on the increased fat accumulation in muscles are required in the future. In addition, the prevalence of diabetes in Group B, C, and D was higher compared with Group A (Table 3). Long duration of diabetes and poor glycemic control are more likely to be associated with microvascular and macrovascular disease [54], and to cause sarcopenia and decrease of MQ independent of BMI and age [54, 55].

The combined index of WC and MQ was well associated with CVD risk factor accumulation in obese patients (Table 4, Fig. 3). Atkins et al. reported a review that summarized studies of the association of CVD risk factors with combinations of various obesity- and sarcopenia-evaluated indices [56]. To the best of our knowledge, however, there have been no reports investigating the association between the accumulation of CVD risk factors and a combined index of WC and MQ, especially in obese patients only. Murai et al. reported that patients with type 2 diabetes who had both VF accumulation and low MQ were more affected with CVD [33]. Boettcher et al. have reported that fat accumulation in the muscles was significantly linked with VF. Therefore, a combined index of WC and MQ may reflect the adverse effects of VF [57]. The effectiveness of the combined index of WC and MQ as a relevant indicator of accumulation of CVD risk factors in obese patients requires further investigation.

The use of multiple medications per day is common with aging. Many drugs taken regularly for diseases may interact with some mechanisms that can alter the balance between protein synthesis and degradation [58], and researchers have reported that polypharmacy is associated with sarcopenia [59]. In this study, diuretics and biguanide (BG) were used significantly more often in Group D compared with Group B and Group A, respectively (Supplementary Table S1). The use of diuretics, particularly loop diuretics, has been suggested as a risk factor of sarcopenia [60]. On the other hand, BG improves insulin resistance and may inhibit the progression of sarcopenia [58]. Unfortunately, we did not assess insulin resistance in this study, but it has been reported that patients with SO are in an insulin-resistant state [1, 3]. It is likely that BG was provided for insulin resistance in Group D in this study. The effects of BG on muscle remain to be elucidated.

The present study had several limitations that warrant mention. First, our study used a cross-sectional design. Thus, we could investigate only the associations between the combined index of WC and MQ and CVD risk factor accumulation. Second, because this study was hospital-based in design and limited to Japanese obese patients, there may be bias among the study participants, which could limit the generalization of the study results. However, our purpose of this study was to identify obese patients at high risk of CVD. In this concept, we were able to identify the particularly high-risk obese patients using the combined index of WC and MQ. Third, the cutoff values for both WC and MQ were not clear. Fourth, we had not been able to accurately assess the amount of physical activity and estimated calorie intake in obese patients. To resolve these limitations, larger cohort and prospective studies including various populations are needed in the future.

In conclusion, our study demonstrated that the obesity-evaluated index, WC, and the sarcopenia-evaluated index, MQ, were most closely associated with CVD risk factor accumulation in Japanese obese patients, respectively. Furthermore, classification by the combined index of WC and MQ reflects CVD risk factor accumulation in Japanese obese patients, regardless of sex and age.

## Supplementary Information

Supplementary Table1

## Data Availability

The data sets used and/or analyzed during the current study are available from the corresponding author on reasonable request.
