# Supplementary material for: A combined index of waist circumference and muscle quality is associated with cardiovascular disease risk factor accumulation in Japanese obese patients: a cross-sectional study
Source: Endocrine. 2022 Apr 19;77(1):30–40. doi: 10.1007/s12020-022-03052-5 (PMC9242950; doi:10.1007/s12020-022-03052-5)
Supplement: Supplementary file 1 — Supplementary Table1 [file 12020_2022_3052_MOESM1_ESM.docx]

**Table S1 Medications of obese patients classified by a combined index of WC and MQ**

|  | Group C (n = 54) | Group S (n = 35) | Group O (n = 41) | Group SO (n = 58) | *p* value^§^ |
| --- | --- | --- | --- | --- | --- |
| Medications for hypertension (n) | |  |  |  |  |
| CA | 18 | 15 | 13 | 18 | 0.669 |
| ACEI | 3 | 3 | 0 | 1 | 0.169 |
| ARB | 9 | 7 | 7 | 13 | 0.863 |
| Diuretics | 4 | 0 | 3 | 11† | 0.017 |
| β | 1 | 2 | 0 | 3 | 0.373 |
| αβ | 0 | 0 | 0 | 2 | 0.210 |
| DRI | 0 | 1 | 0 | 0 | 0.222 |
|  |  |  |  |  |  |
| Medications for diabetes (n) | |  |  |  |  |
| SU | 4 | 4 | 4 | 10 | 0.417 |
| DPP4I | 4 | 7 | 9 | 15 | 0.077 |
| BG | 2 | 6 | 6 | 13* | 0.041 |
| SGLT2I | 4 | 4 | 4 | 9 | 0.581 |
| GLI | 0 | 1 | 0 | 0 | 0.222 |
| αGI | 1 | 0 | 1 | 0 | 0.581 |
| Insulin | 2 | 0 | 0 | 5 | 0.078 |
|  |  |  |  |  |  |
| Medications for dyslipidemia (n) | |  |  |  |  |
| Statin | 23 | 17 | 10 | 17 | 0.073 |
| Fibrate | 1 | 1 | 1 | 0 | 0.483 |
| ω3 | 7 | 5 | 4 | 9 | 0.866 |

CA, calcium channel antagonist; ACEI, angiotensin converting enzyme inhibitor; ARB, angiotensin receptor blocker; β, β-blockade; αβ, αβ-blockade; DRI, direct renin inhibitor; SU, sulfonyl urea; DPP4I, dipeptidyl peptidase–4 inhibitor; BG, biguanide; SGLT2I, sodium glucose cotransporter 2 inhibitor; GLI, glinide; αGI, alpha glucosidase inhibitor.

^§^ *p* value for difference among four groups (chi-square test).

* *p* < 0.05 vs. group C; † *p* < 0.05 vs. group S.
